# Trastuzumab resistance in HER2-positive breast cancer: Mechanisms, emerging biomarkers and targeting agents

**DOI:** 10.3389/fonc.2022.1006429

**Published:** 2022-10-06

**Authors:** Zhen-hao Wang, Zhuo-qun Zheng, Shi−cheng Jia, Shu-ni Liu, Xiao-fen Xiao, Guan-yuan Chen, Wei-quan Liang, Xiao-feng Lu

**Affiliations:** ^1^ Department of Thyroid and Breast Surgery, Clinical Research Center, The First Affiliated Hospital of Shantou University Medical College (SUMC), Shantou, China; ^2^ Shantou University Medical College (SUMC), Shantou, China; ^3^ Guangdong Provincial Key Laboratory for Diagnosis and Treatment of Breast Cancer, Shantou, China

**Keywords:** breast cancer, HER2-positive, trastuzumab, resistance, mechanism, biomarker, targeting agent

## Abstract

Trastuzumab is a standard molecular targeted therapy for human epidermal growth factor receptor 2(HER2) -positive breast cancer, which can significantly improve the survival of patients with this molecular subtype of breast cancer. However, the clinical problem of onset or secondary resistance to trastuzumab has limited its efficacy. Therefore, it is very important to explore the mechanism of trastuzumab resistance and formulate countermeasures. Our study described the underlying molecular mechanism of trastuzumab resistance including *ERBB2* mutations and nuclear localization, transcriptional and post-translational alterations of *ERBB2*, over-activation of bypass signaling pathways activation and so on. Then summarize the potential emerging predicting biomarkers and therapeutic strategies for trastuzumab resistance, in order to provide research direction for reversing trastuzumab resistance.

## 1 Introduction

The International Agency for Research on Cancer (IARC) released statistics that show breast cancer has overtaken lung cancer as the most common cancer worldwide.at the end of 2020 (1). Among several subtypes of breast cancer classified by molecular characteristics, HER2-positive tumors account 15% to 20% of all breast cancers and display more aggressive behaviors and a poorer prognostic clinical course (2). Among targeted therapy for patients with HER2-positive breast cancer, trastuzumab (Herceptin) serves as the first FDA-approved and most common drug. However, About 70% of HER2-positive breast cancer patients, indeed in spite of the fact that they at first react to trastuzumab or trastuzumab conjugate regimens, create resistance inside one year of treatment start (3), indicating that although introduction of anti-HER2-based trastuzumab represents a striking advance, resistance to trastuzumab therapy leading to recurrence still limits the survival of HER2-positive breast cancer patients. Accordingly, understanding the molecular mechanisms of trastuzumab resistance will have a significant impact on the ability to cure HER2-positive breast cancer. This article analyzes the recent research on the molecular mechanisms behind trastuzumab resistance and provides a niche strategy for potential, molecular targets to overcome resistance to trastuzumab.

## 2 Currently investigated mechanisms related to trastuzumab resistance

### 2.1 *ERBB2* mutations and nuclear localization are associated with trastuzumab resistance

Previous studies have revealed that *ERBB2* mutations can drive various carcinomas. HER2 receptors comprise of an extracellular ligand-binding space, a transmembrane space and an intracellular space. Mutations have been observed in each domain, but overall, *ERBB2* mutations occur in less than 5% of breast cancer patients. Interestingly, *ERBB2* mutations rarely co-exist with *ERBB2* amplification (4). Moreover, based on the results of both DNA and RNA sequencing information from an assortment of solid tumors splices (n=5151), studies have revealed that *ERBB2* alterations co-exist with mutations of *ERBB3, RAF1*, and *PIK3CA*, suggesting that both MAPK and PI3K pathways are essential for tumorigenesis mediated by mutated *ERBB2 (* 5). Previous studies have indicated that the progression-free survival (PFS) of patients with *ERBB2* mutations is usually shorter than in patients with wild-type *ERBB2*, suggesting that *ERBB2* mutations may increase the resistance to chemotherapeutic agents (6). However, the exact mechanism remains unclear (7, 8).

Over-expression of membrane HER2 receptor tyrosine kinase plays an indispensable part in breast cancer. Nevertheless, current anti-HER2 remedies, as with the antibody trastuzumab, target membrane HER2 receptor only. Studies have reported that nuclear *HER2* interacts with *HER3* at the cyclin D1 promoter to form a transcriptional compound body, driving cyclin D1 expression and activating cell cycling, which cannot be suppressed by trastuzumab. Also, inhibition of the HER2/HER3 dimers reduces the progression of breast tumors (9). Since trastuzumab only targets membrane HER2, the cell proliferative effect triggered by nuclear HER2 complexes may account for much of the development of trastuzumab resistance (10, 11).

### 2.2 Transcriptional alterations of *ERBB2* account for trastuzumab resistance

*d16-HER2* is a splice variant derived from exon 16 skipping in *ERBB2*, and is expressed in many HER2-positive mammary tumors. *d16-HER2* is supposed to constitutively generate activated d16-HER2 homodimers on the tumor cell surface that phosphorylate and activate SRC (pSRC) kinase to enhance cell proliferation and tumorigenesis. Mitra *et al.* observed that d16-HER2 strongly activates multiple oncogenic pathways and amplifies the transference and invasion in MCF-7 cells compared to wild-type HER2 (22). Studies have also observed that the expression of d16-HER2 protein in healthy mice accelerates mammary tumorigenesis. In all, *d16-HER2* may increase the tumorigenesis of breast cancer cells and account for poor sensitivity of breast tumors to trastuzumab (23, 24).

### 2.3 Post-translational alterations of *ERBB2* are related to trastuzumab resistance

About 30% of HER2-positive breast tumors express a group of truncated HER2 segment collectively named as p95-HER2, and also known as the p95-HER2/611 carboxy terminal fragment (CTF) and p110 (25). p95-HER2 is a membrane-related truncated isoform of HER2, devoid of its extracellular domain and trastuzumab epitope, which may play a role in trastuzumab resistance (26, 27). It is believed that p95-HER2 is a poor prognostic factor for HER2-amplified breast cancers. Chumsri *et al.* discovered that a high p95-HER2/HER2 ratio is associated with poor prognosis in patients with HER2-positive metastatic breast cancer (28). However, some studies repudiated, arguing that increasing of p95-HER2 may benefit in trastuzumab therapy. Sperinde J *et al.* reported that DDFS (distant disease-free survival) in the arm receiving chemotherapy only was shorter than those who received chemotherapy plus trastuzumab (29). The exact impacts of p95-HER2 on trastuzumab therapeutic effect are still remain unclear and warrant further investigation.

### 2.4 Over-activation of signaling pathways results in trastuzumab resistance

The fact is well known that abnormal activation of the PI3K/AKT/mTOR pathway dominates the carcinogenesis of breast cancer, and increases resistance to trastuzumab (30, 31). The *PIK3CA* regulates the formation of PI3K, one of the most commonly mutated genes in breast cancer. *PIK3CA* over-activates the PI3K/AKT/mTOR pathway and accelerate the transformation and metastasis of mammary epithelial cells, resulting in trastuzumab resistance (32). The SRC oncogene is also involved in various tumor signaling pathways, and is often abundantly expressed in breast cancer. Activation of SRC can result in activation of a variety of downstream molecules, such as AKT, ERK and STAT3, and enhance the PI3K/AKT signaling pathway to lead to the results in increased cell proliferation, survival, motility, and angiogenesis (33).

**Figure 1:**
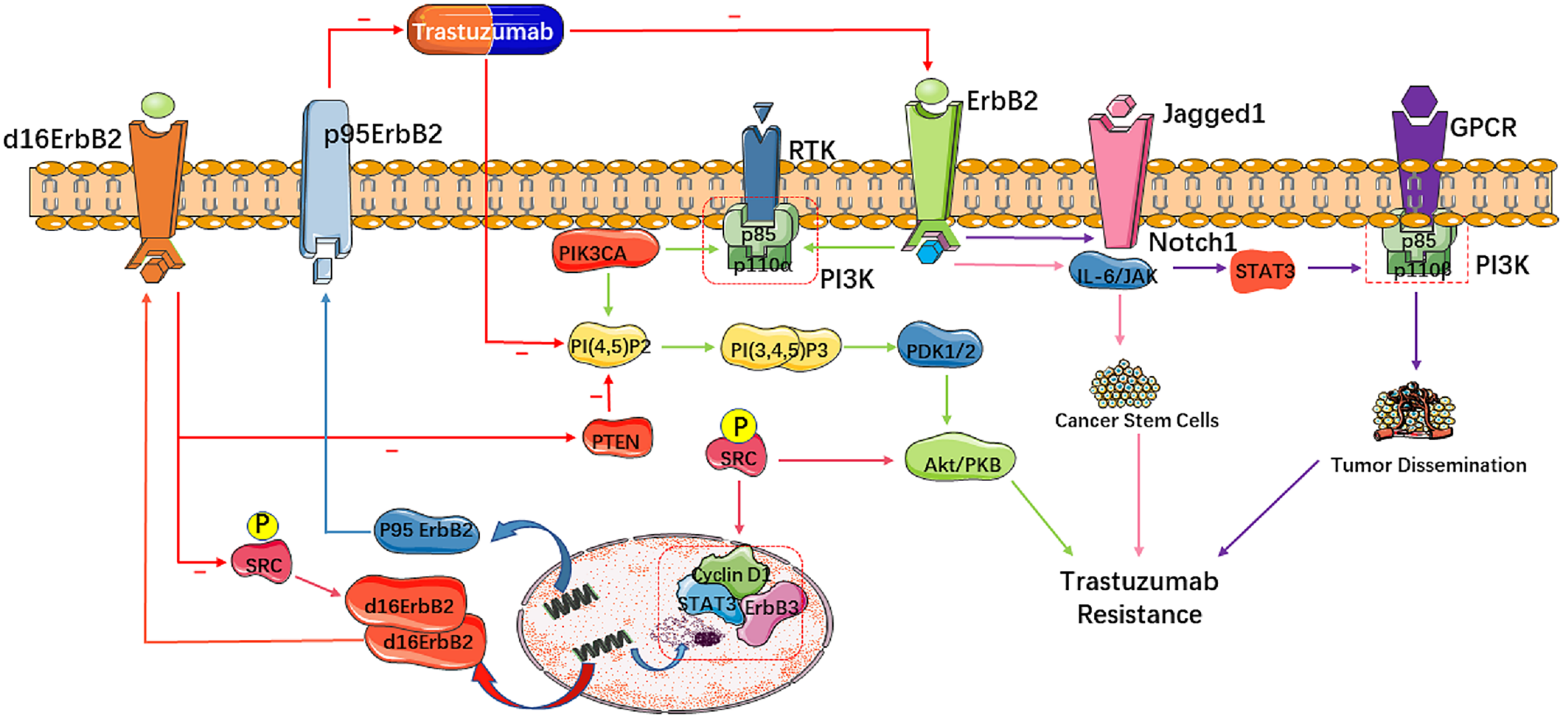
Molecular mechanisms of trastuzumab resistance in HER2-positive breast cancer. These molecular mutations mainly impact cell signaling and proliferation, leading to trastuzumab resistance. Alterations of HER2 influencing downstream pathways abate the pharmacologic effect of trastuzumab. A truncated form of HER2 called p95-HER2 lacks the extracellular domain that binds trastuzumab and is related with poor response to trastuzumab. d16-HER2, derived from exon 16 skipping in *ERBB2*, generates activated d16-HER2 homodimers on the tumor cell surface to activate SRC kinase, resulting in cell proliferation and tumorigenesis.

In addition to activating the PI3K/AKT/mTOR pathway directly, HER2 may also contribute to trastuzumab resistance towards breast tumors through aberrant activation of other signaling pathways. The Notch family is a well-known and essential regulator of HER2-positive cancer stem cells (CSCs) (34). Studies have shown that treatment with trastuzumab downregulates HER2 receptors and leads to an increase of membrane Jagged 1 and activation of the Notch receptor, resulting in the survival and renewal of CSCs (35, 36). CSCs initiate and maintain the growth, progression and metastasis of tumors, which are regulated by the Notch family through the STAT3 pathway. The activation of STAT3 then initiates downstream proliferative and anti-apoptotic pathways involving G protein-coupled receptors (GPCRs) and Toll-like receptors (TLRs), as well as interleukin-6 (IL-6) and its familial members, ultimately leading to trastuzumab resistance in HER2-positive breast tumors (21, 35, 36). Moreover, STAT3 activation may also contribute to the development of trastuzumab resistance byupregulating the genes that encodes cell cycle regulators (cyclin D1, c-Myc), Inhibitors of apoptosis (Bcl-2, survivin) and angiogenesis-inducing factors (HIF-1α, VEGF) (37). Overall, trastuzumab induces its own resistance through activating the Jagged-1/Notch/STAT3 signaling pathway, as well as promoting cell proliferation and angiogenesis (**Figure 1**).

## 3 Biomarkers for predicting trastuzumab resistance

### 3.1 Bypass activation

Several molecules have been identified that may contribute to the progress of primary or secondary resistance to trastuzumab *via* the activation of bypassing signaling pathways, and could serve as potential as biomarkers for predicting trastuzumab resistance (**Table 1**).

**Table 1:** Emerging biomarkers for predicting trastuzumab resistance through bypass pathway activation.

Biomarkers	Mechanism/signaling pathway	Primary/secondary drug resistance	Correlation with drug resistance	Author and reference
**CDK12**	WNT/β-catenin/-TCF; IRS1/ErbB/PI3K/AKT	Primary and Secondary	Positive	Choi HJ (12)
**Cullin7**	IRS-1/ErbB/PI3K/AKT	Primary and Secondary	Positive	Qiu N (13)
***ARID1A***	ErbB/PI3K/AKT	Primary and Secondary	Negative	Berns K (14)
**STAT3**	IL6-pSTAT3-PTEN	Primary	Positive	Sonnenblick A (15)
***NCAPG***	JAK/STAT3	Primary	Positive	Jiang L (16)
***ANKRD44***	TAK1/Akt/NF-kβ	Secondary	Negative	La Ferla M (17)
**MUC4**	NF-κB/MUC4	Primary	Positive	Mercogliano MF (18)
**YAP1**	Hippo/YAP1/TEAD	Secondary	Positive	González-Alonso P (19)
**EPHA5**	Notch1; PTEN/AKT	Primary	Negative	Li Y (20)
**PKCα**	Jagged-1/Notch-1	Primary	Negative	Pandya K (21)

#### 3.1.1 The PI3K/AKT pathway

Cyclin-dependent kinase 12 (CDK12), located on chr17q12, has a high concurrent amplification rate similar to HER2 (about 90% of HER2+ breast cancers), and the results show that CDK12 enhances CSC self-renewal in breast cancer in a manner independent of HER2. The ability of CDK12 kinase to phosphorylate and activate the RNA Pol II CTD is up-regulated by transcription of WNT ligands and IRS1. To maintain breast CSCs, WNT/β-catenin/TCF signaling can promote HER2-bypassing signal transduction through IRS1-mediated ErbB-PI3K-AKT signaling, thereby inducing tumor resistance to trastuzumab. The results show that CDK12 amplification was found to be a potential predictor of trastuzumab response, and suggest HER2-positive breast cancer patients with simultaneous amplification of HER2 and CDK12 can benefit from CDK12 kinase inhibition (12).

Cullin7 (Cul7) belongs to the Cullin family and acts as a scaffold protein for E3 ubiquitin ligase which binds the small RING finger protein ROC1, Skp1, and F-box protein Fbw8. Evidence shows that Cul7 mediates the degradation of the serine phosphorylation-inhibited IRS-1, leading to the removal of IRS-1 with a blocked tyrosine phosphorylation site, thereby enabling IRS-1-mediated PI3K/AKT signal transduction, induced cancer stem cell-like features and drug resistance to trastuzumab. This study suggests that Cul7 can be used as a biomarker to predict trastuzumab sensitivity (13).

It has been suggested that SWI/SNF subunits may act as tumor suppressors in cancer, and ARID1A is the most commonly mutated SWI/SNF gene. Berns *et al.* found that the loss of the ARID1A gene could activate the expression of Annexin A1 (ANXA1), and then activate the AKT pathway to enable breast cancer cells to obtain trastuzumab resistance during adjuvant therapy. So, the level of ANXA1 can also predict the sensitivity to trastuzumab in targeted therapy for breast cancer (14).

#### 3.1.2 The STAT3 pathway

As mentioned above, STAT3 has been found to be an important part of many carcinogenic signaling pathways, and it is also closely related to the mechanism of trastuzumab resistance. Sonnenblick *et al.* have shown that there is a potential link between the loss of IL6-pSTAT3-PTEN, matrix activation and the generation of primary trastuzumab resistance in HER2-positive primary breast cancer. A combination of IL6-STAT3 inhibitors and trastuzumab may be effective for treating primary HER2-positive breast cancer (particularly in patients lacking PTEN). This also indicates that STAT3 could be used as a biomarker to predict the sensitivity to trastuzumab (15).

In addition, Jiang *et al.* found that the NCAPG gene reverses trastuzumab’s effects on HER2-positive breast cancer cells by activating the JAK/STAT3 signaling pathway. Moreover, knockdown of NCAPG can resensitize HER2-resistant breast cancer cells to trastuzumab, indicating that NCAPG has the potential to become a predictive biomarker and target for therapeutic intervention for trastuzumab resistance in HER2-resistant breast cancer cells (16).

#### 3.1.3 The NF-κB pathway

La Ferla *et al.* found that ANKRD44 gene silencing up-regulated the expression of the TAK1/Akt pathway, leading to constitutive activation and nuclear accumulation of the NF-kβ p65 subunit, thereby inducing acquired trastuzumab resistance. At the same time, this process was closely related to an increase in glycolysis. These results show that the ANKRD44 gene has the potential to become one of the predictive biomarkers of acquired trastuzumab resistance (17).

A glycoprotein called mucin 4 (MUC4) is a membrane-binding mucin composed of two non-covalently bound subunits that are encoded by a single gene. Mercogliano *et al.* found that TNFα (tumor necrosis factor-α) is an important cytokine that induces MUC4 expression so as to mediates resistance to trastuzumab in HER2-positive breast cancer cells. In HER2-positive human breast cancer and gastric cancer cells, trastuzumab resistance is induced by high expression of MUC4 through the NF-κB pathway to reduce the effectiveness of ADCC (antibody-dependent cell-mediated cytotoxicity) in breast cancer cells and then induce drug resistance. On the one hand, MUC4 is a glycoprotein of the mucin family that has been found to be capable of detecting HER2-positive breast cancer response to trastuzumab in a specific manner. On the other hand, TNFα blockers can inhibit the expression of MUC4 and then overcome the resistance of primary trastuzumab in HER2-positive breast cancer, and may also be used as a potential new therapeutic target (18).

#### 3.1.4 The Hippo-yap pathway

Increasing evidence shows that the Hippo pathway can regulate primary and acquired resistance to anti-cancer therapy. González-Alonso *et al.* have demonstrated that when the Hippo pathway is inactivated, YAP1 is dephosphorylated and transported to the nucleus, and then combines with the transcription factor TEAD to mediate acquired trastuzumab resistance by promoting tumor cells proliferation. Thus, YAP1 protein can be used as one of the biomarkers for predicting acquired resistance in trastuzumab therapy (19).

#### 3.1.5 The Notch1 pathway

EPHA5 belongs to the Eph receptor family. Studies have found that EPHA5 is a candidate for cancer diagnosis and prognosis prediction. Moreover, EPHA5 is closely related to dormant cells with stem cell-like characteristics. Li *et al.* found that EPHA5 deficiency promotes Notch1 and PTEN/AKT pathway activation to induce trastuzumab resistance, and is related to the number and performance of breast cancer stem cells, suggesting EPHA5 as a potential biomarker for predicting primary resistance to trastuzumab (20).

The ErbB-2 pathway is also mediated by Protein kinase C-α (PKCα). Pandya *et al.* found that PKCα reduced the binding between Jagged-1 and Mib-1 (Ki-67) to limit the ubiquitination of Jagged-1 and Jagged-1-mediated transactivation of Notch-1, thereby preventing Notch-1-driven drug resistance to anti-HER2 targeted therapy. On the one hand, this shows that PKCα protein levels could be used as a potential biomarker for predicting sensitivity to trastuzumab. On the other hand, the dual targeting of HER2 and Notch can prevent recurrence and partially reverse the resistance to trastuzumab. Therefore, the therapy targeting Jagged-1 may reverse trastuzumab resistance by reducing Notch activity (21).

As for the progress of related signaling pathways in the treatment of HER2-positive breast cancer, the clinical research on overcoming trastuzumab resistance through the PI3K inhibitor alpelisib has made progress to a certain extent, a phase III clinical trial is ongoing evaluating alpelisib in combination with trastuzumab for patients with HER2-positive MBC and PI3K mutations(NCT04208178). Meanwhile, the development of therapeutic drugs targeting other signaling pathways is currently in need of more clinical trials for overcoming trastuzumab resistance.

### 3.2 MicroRNAs for predicting trastuzumab resistance

Current studies have demonstrated that microRNAs are essential for both expression of HER2 and the action of trastuzumab in breast cancer cells. Heterodimerization of HER2 with other HER family members under the regulation of miRNAs could induce a variety of intracellular signaling pathways and induce trastuzumab resistance in breast cancer cells. Based on this, miRNAs not only can be utilized as significant predictors of trastuzumab resistance in HER2-positive breast cancer cells, but also can guide the development and application of targeted drugs. The former, such as miRNA-125a-5p and miRNA-199b-5p, are prominent tumor suppressors in breast cancer, but the specific mechanism is not yet clear (38, 39). In contrast, reduced ectopic expression of miRNA-221 can downregulate PTEN expression and lead to trastuzumab resistance in breast cancer cells (40, 41). All of these factors could be used to predict the development of drug resistance in HER2-positive breast cancer cells. A more common mechanism for the latter is that miRNAs regulate trastuzumab resistance *via* HER2 surrogate receptors. For example, miR-128-3p and miR-30a-5p regulate the IGF-1R/Akt/mTOR pathway, interfering with the FOXO3a-miRNA axis and thus inducing resistance (42). In addition, miR-126 acts as a pivotal part in the regulation of the Akt/mTOR pathway. As demonstrated by Fu *et al.*, low expression of miR-126 could facilitate tumor cell motility and invasion. Once miR-126 is overexpressed, sensitivity to trastuzumab could be restored and the ability of tumor cells to invade and migrate inhibited. The authors also concluded that this phenomenon is based on PIK3R2-dependent activation of the PI3K/AKT/mTOR signaling pathway. Generally speaking, these findings pave the way for application of microRNA as a tool against resistance to trastuzumab in breast cancer (43).

### 3.3 Tumor microenvironment markers for prediction of trastuzumab resistance

In addition to genetic and epigenetic changes in cells, the tumor microenvironment also plays a role in tumor cells acquiring resistance, where tumor cells actively recruit stromal cells from the microenvironment into the tumor, while secreting soluble stromal regulatory factors to modify these stromal cells, and thus provide a microenvironment that supports tumor progression. Fernández-Nogueira *et al.* discovered that tumor fibroblasts could induce tumor cell resistance to trastuzumab in HER2-positive breast cancers, and they found that HER2-positive TAFs (tumor-associated fibroblasts) secreted FGF5 (fibroblast growth factor 5), which connects to fibroblast growth factor receptor 2 (FGFR2) to transactivate HER2 by phosphorylating the *c-Src* proto-oncogene. This in turn induces resistance to trastuzumab and lapatinib, a reaction related to the mechanism by which FGFR2 inhibitors suppress tumor progression in trastuzumab-resistant and lapatinib-resistant cells and restore sensitivity to HER2-targeted therapies (44). In addition, studies have demonstrated that FGF5 levels in the stroma can be a method for predicting efficacy of trastuzumab in treating HER2-positive breast cancer patients.

Vathiotis *et al.* Analyzed the expression of proteins in patients treated with trastuzumab with univariate statistics. An association between short DFS(p = 0.002) and high expression of smooth muscle actin (α-SMA) was identified in the stroma, suggesting that quantification of α-SMA in the cell stroma could be a novel and easily implemented method to predict trastuzumab resistance (45).

### 3.4 Molecules carried by exosomes predict trastuzumab resistance

Exosomes execute their function as extracellular vesicles. Once released and secreted from the host cell, exosomes target a recipient cell to regulate the biological activity of the recipient cell through the transfer of their encapsulated proteins, nucleic acids, and lipids. Exosomes are increasingly being studied as exclusive information media of the tumor microenvironment and have recently been found to be linked to metastatic drug resistance in tumors. Martinez *et al.* was able to distinguish exosome-carried transforming growth factor-β1 (TGFβ1) from serum TGFβ1 for identification of patients who would gain benefit from HER2-targeted therapy (trastuzumab). They found that HER2-positive breast cancer cells express neuromedin U(NmU), which blocks the immune response and promotes the expression of immunosuppressive molecules, such as TGFβ1., therefore functionally affecting ADCC and inducing trastuzumab resistance. Thus, detection of exosome- associated TGFβ1 levels could be a potential new method for predicting therapeutic response to trastuzumab-targeted therapy in HER2-positive breast cancer (46, 47).

In addition, a number of miRNAs can be transferred by exosomes to induce resistance and advance progression in cancer cells (48). Han *et al.* showed miR-567 could reverse trastuzumab resistance in breast cancer. The authors showed a reduction in miR-567 is associated with trastuzumab resistance and that downregulation of miR-567 induces resistance to trastuzumab. Of interest, incorporating miR-567 into exosomes and targeting ATG5 allowed miR-567 to overcome trastuzumab resistance through exosome delivery. In conclusion, exosome miR-567 plays a important role in overcoming trastuzumab resistance, suggesting that it may serve as a useful target of therapeutic interventions and prognostic indicator for breast cancer patients (49). Liu *et al.* found that targeting the 17q23 amplicon could also promote trastuzumab resistance, in HER2-positive breast cancer in a manner dependent on the upregulation of miR-21. Inhibition of miR-21 selectively inhibited the survival, proliferation and oncogenic potential of HER2-positive breast cancer cells carrying the 17q23 amplicon and suppressed the emergence and spread of trastuzumab resistance in breast cancer cells (50). This result implicated the great potential of miR-21 inhibition coupled with other medication to target trastuzumab-resistant HER2-positive breast cancer.

## 4 Novel therapeutic agents against trastuzumab resistance

### 4.1 Tyrosine kinase inhibitors

Poziotinib inhibits pan-HER kinases irreversibly as an oral medication. Poziotinib monotherapy was evaluated in a phase II study conducted on patients with HER2-positivity who had received two or more HER2-directed therapies in the past. There was a 12-month median follow-up, and the median PFS was 2.94 to 4.40 months with a 95% confidence interval of 2.94 to 4.40 months for overall survival. In terms of adverse events related to the treatment, diarrhea was the most common (96.23%/14.15%), followed by stomatitis (92.25%/12.26%) and rash (63.21%/3.77%). These heavily treated HER2-positive mBCs responded significantly to Poziotinib (51).

Epertinib (S-222611) is a powerful EGFR/HER2 inhibitor that has recently been approved by the FDA. An extended phase Ib study found a 19.0% objective response rate (ORR) in patients with heavy pretreatment HER2-positive breast cancer., and An individual with brain metastasis had a 7.5-month partial response (52). The TKIs mentioned above were tolerable and demonstrated meaningful antitumor properties in patients with heavily pretreated HER2-positive breast cancer, including those with brain metastases.

### 4.2 Antibody-drug conjugates

Trastuzumab-deruxtecan (DS-8201a, T-DXd) is another novel antibody drug conjugate (ADCs) after trastuzumab emtansine (T-DM1). In the DESTINY-Breast01 trial, all patients that participated had been treated with trastuzumab and T-DM1, 66% had been treated with Pertuzumab, and in excess of half with HER2 therapeutics, examples include lapatinib. In the same population, 60.9% of patients showed clinically relevant response rates, with virtually all showing some shrinkage of tumors; There was a median PFS of 16.4 months (95% CI 12.7-NE) and no median OS was available. As defined by complete or partial response over six months, 76.1% (95% CI 69.3-82.1) of persons responded, and the median duration was 14.8 months (95% CI 13.8-16.9) of persons responded. For patients with brain metastases enrolled into the trials, the PFS was comparable to that for overall population (median PFS 18.1 months; 95% CI 6.7–18.1 months). And the results from the DESTINY-Breast02 trial in this advanced disease patient population confirm the efficacy and safety of T-DXd (53). In the DESTINY-Breast03 trial, in patients who have previously received trastuzumab and taxanes, there was a lower risk of disease progression or death among those treated with trastuzumab deruxtecan compared to those treated with trastuzumab emtansine (54). In addition, it has been reported in DESTINY-Breast04 that preliminary studies with T-DXd in patients with breast cancer expressing low levels of HER2(Immunohistochemical [IHC] staining of HER2 was defined as 1+ or 2+ and negative *in situ* hybridization results were defined as low expression of HER2) have shown promising effects, widening the scope of application of HER2 targeted therapy (55, 56), it is worth mentioning that this study breaks the binary pattern of anti-HER2 therapy.

Trastuzumab duocarmazine (SYD985) is also currently making good progress. In 2018, Xu Z *et al.* reported drug safety data from 50 patients with HER2+ breast cancer, 80% of whom got remedy of T-DM1. According to preliminary results, SYD985 had a 33% objective response rate (ORR) and a median progression-free survival (PFS) of 9.4 months. There was a 27% ORR among HER2-low metastatic breast cancers, including hormone receptor-positive (N = 32) and triple-negative (N = 17) breast cancers. Most commonly encountered adverse events (AEs) were fatigue, dry eyes, conjunctivitis, and increased tears. Gradually significant adverse events (AEs) included neutropenia (6%) and conjunctivitis (4%) (57). In summary, T-DXd and SYD985 are emerging as new options for advanced HER2+ patients who have become resistant to HER2-targeted therapy.

### 4.3 Other novel agents

p95HER2-TCB, a bispecific antibody that attaches to p95-HER2 protein-expressing breast cancer tumor cells and T lymphocytes, guides the immune system to target and kill HER2-positive breast cancer cells. In terms of efficacy and safety have been investigated in cellular assays and *in vivo* experiments in mice, thus completing the preclinical development phase. This new anti-tumor immune drug had a potent antitumor effect on p95HER2-expressing primary breast cancer and brain lesions (48). Another novel bispecific antibody, ZW25, binds simultaneously to two HER2 epitopes: ECD4, which is the trastuzumab domain of binding, and ECD2, which is the pertuzumab domain of binding. Preclinical studies have shown that The effectiveness of ZW25 in treating tumors with HER2 expression levels across a wide range may be greater than that of trastuzumab over a broad range in silencing HER2 signaling, but specific efficacy and drug safety require further demonstration in clinical studies (58).

Margetuximab is an immunoglobulin G1 (IgG1) monoclonal antibody that activates the immune system against ERBB2 that contains the same epitope specificity and antiproliferative effects as trastuzumab without the Fc component. Clinical trial results from a phase III randomized trial showed a valid increasing in PFS (median PFS of 5.7 months versus 4.4 months) and a 24% reduction in relative risk (RR) in the margetuximab plus chemotherapy arm compared to the trastuzumab plus chemotherapy arm in advanced HER2-positive breast cancer patients (after receiving targeted therapy previously), with a median overall survival (OS) of 21.6 months, compared with a median OS of 19.8 months in the trastuzumab group, and the regimen also had an acceptable safety profile (59).

In summary, the success of clinical research and the improved understanding of the biological heterogeneity of HER2-positive cancer over the years, several approaches in early and metastatic treatment have been developed important choice to overcome trastuzumab resistance. Many new drugs, including drugs targeting p95HER2 and immunoconjugates, are expected to improve the status in the comprehensive treatment of her2-positive breast cancer.

## 5 Conclusions

At present, trastuzumab resistance stresses the need for more potential therapeutic targets on the basis of dual-target or even multi-targeted therapy. Developing a deeper better understanding of these drug resistance-related mechanisms will enable the identification of promising potential therapeutic targets for formulating effective strategies to overcome drug resistance. In this paper, the drug resistance mechanisms of trastuzumab identified at present can be summarized as principally HER2 gene mutations, post-translational alterations of *ERBB2* and activation of related signaling pathways.

In an era of individualized precision medicine, breast cancer is currently clinically staged by the major biomarkers of ER, PR, Ki67 and HER2, with HER2 status being the gold standard for the use of trastuzumab-targeted therapy (60). Consequently, high-quality resistance-related markers are needed to form the basis for formulating precise and cost-effective treatment strategies. Although most biomarkers have not yet been approved for clinical application, it is undeniable that the prospect of biomarkers still needs focus. Effective predictive biomarkers, on the one hand, can be used to predict high-risk groups for timely adjustment of treatment plans, and on the other hand, can be useful for accurately separating patients who would benefit only from monotherapy from those who require combination chemotherapy, to guarantee favorable prognosis and effectively reduce the cost of treatment, as well as diminish the side effects of chemotherapy to some degree. At present, among the biomarkers for trastuzumab resistance, in addition to some upstream factors of some classical signaling pathways, miRNAs and exosome-related molecules in the tumor microenvironment have potential, and more research is needed to verify their clinical significance and explore suitable detection methods, such as circulating tumor DNA.

Currently, although there are many emerging therapies for HER2-positive breast cancer, including TKIs, ADCs, and bispecific antibodies, trastuzumab is still irreplaceable in the treatment of HER2-positive breast cancer. The investigation of trastuzumab resistance mechanisms and their associated markers remains a top priority. In this paper, we believe that research on mechanisms of trastuzumab resistance, emerging biomarkers and targeted agents will lay the foundation for additional strategies for targeted therapy in HER2-positive breast cancer.

## Author contributions

X-FL and W-QL conceived and designed the project. S-CJ, S-NL, X-FX and G-YC analyzed the data and prepared the figures. Z-HW and Z-QZ wrote the manuscript. X-FL and W-QL approved the final version to be submitted. All authors contributed to the article and approved the submitted version.

## Funding

This study is partly supported by Interdisciplinary project of Li-Ka-Shing Foundation, (No.2020LKSFG05C)Natural Science Foundation Committee (82102948), Natural Science Foundation of Guangdong Province (2021A1515011180 and 2019A1515010239), Guangdong Basic and Applied Basic Research Foundation (No. 2019A1515110953), General projects of Chinese Postdoctoral Science Foundation(2020M672753), Guangdong Science and technology Special Fund project (210715106900933); Guangdong Provincial Key Laboratory for Breast Cancer Diagnosis and Treatment (2017B030314116).

## Conflict of interest

The authors declare that the research was conducted in the absence of any commercial or financial relationships that could be construed as a potential conflict of interest.

## Publisher’s note

All claims expressed in this article are solely those of the authors and do not necessarily represent those of their affiliated organizations, or those of the publisher, the editors and the reviewers. Any product that may be evaluated in this article, or claim that may be made by its manufacturer, is not guaranteed or endorsed by the publisher.
